# Interdigitated Sensor Optimization for Blood Sample Analysis

**DOI:** 10.3390/bios10120208

**Published:** 2020-12-16

**Authors:** Julien Claudel, Thanh-Tuan Ngo, Djilali Kourtiche, Mustapha Nadi

**Affiliations:** Institut Jean Lamour, Lorraine University (CNRS—UMR 7198), 54011 Nancy, France; julien.claudel@univ-lorraine.fr (J.C.); ngothanhtuan@humg.edu.vn (T.-T.N.); djilali.kourtiche@univ-lorraine.fr (D.K.)

**Keywords:** biosensors, interdigitated electrodes, impedance spectroscopy, blood analysis

## Abstract

Interdigitated (ITD) sensors are specially adapted for the bioimpedance analysis (BIA) of low-volume (microliter scale) biological samples. Impedance spectroscopy is a fast method involving simple and easy biological sample preparation. The geometry of an ITD sensor makes it easier to deposit a sample at the microscopic scale of the electrodes. At this scale, the electrode size induces an increase in the double-layer effect, which may completely limit interesting bandwidths in the impedance measurements. This work focuses on ITD sensor frequency band optimization via an original study of the impact of the metalization ratio *α*. An electrical sensor model was studied to determine the best *α* ratio. A ratio of 0.6 was able to improve the low-frequency cutoff by a factor of up to 2.5. This theoretical approach was confirmed by measurements of blood samples with three sensors. The optimized sensor was able to extract the intrinsic electrical properties of blood in the frequency band of interest.

## 1. Introduction

Many improvements in the sensitivity and selectivity of biosensors have been made in the last two decades [1,2,3,4,5]. They are mainly due to the sensors (lab-on-a-chip) designed in micro-nanotechnology facilities. For biological applications [6], charge transfer sensors [7], impedance-based sensors [8,9,10], and capacitance-based sensors are often used. Impedance spectroscopy is a well-known and powerful technique for biological characterizations on both the macroscopic and microscopic scales [11]. The electrodes apply an electric field to the sample being tested and measure electrical signals. They also can provide information on the relative permittivity and electrical conductivity of biosamples that correspond to intrinsic parameters [12]. Impedance-based sensors are divided into four main categories: cell trap sensors, cytometric sensors, matrix sensors and interdigitated sensors. The sensors in the first three categories have the best sensitivities because only one cell is analyzed at a time. Trap sensors often use micro-hole or microcavity [13] systems to isolate cells from each other. Cytometric sensors use microchannels to focus on one cell at a time [14], and cells are dynamically characterized during their passage into a measurement area situated inside the channel. Matrix electrode systems use multiple electrodes to perform numerous measurements at a time [15]. Despite the fact that these three techniques have better sensitivities, they are also the most difficult to implement. Thus, interdigitated (ITD) sensors [16], composed of just one layer of coplanar electrodes, remain competitive for low-volume and low-concentration biological samples. For example, they are perfectly suitable for cell surface cultures [17] and low-volume biological sampling, as in DNA analyses [18]. Beyond the simple interface, the geometrical properties of the electrodes can have a significant impact on the efficiency of the biosensor [19,20]. They can be optimized a priori during the sensors’ design step according to the targeted application and the nature of the cells to be analyzed [14]. In this work, we propose a method for optimizing the ITD sensor frequency band involving the metalization ratio.

First, we propose a complete electrical equivalent model that takes into account all the sensor’s parameters, such as the electrode length, gap, width, and electrical properties of the medium. Interface capacitive effects, also known as the double-layer effects, are also modeled to assess the impact on the global impedance spectrum.

In the second part, analytical simulations are performed to analyze the effects of geometrical parameters on bioimpedance measurements. It is important to maintain a sufficiently wide bandwidth in order to be able to characterize a biosample over many decades. To achieve optimization, the impact of the metalization ratio *α* on the bandwidth was studied. This ratio is defined as *α = W*/(*S + W*), where *G* is the gap (m) and *W* is the width (m) of the IDT sensor digits (see Section 2.1 below).

The last section focuses on the experimental validation of the two previous sections. Three sensor designs with different degrees of optimization corresponding to different values of the ratio α were fabricated using a standard microfabrication process. Characterizations were performed in calibrated electrolytic solutions to validate our model. Finally, the capability of our optimized sensor in terms of characterizing a blood sample was tested.

## 2. Theoretical Considerations

### 2.1. Sensor Structure and Cell Factor

**Figure 1:**
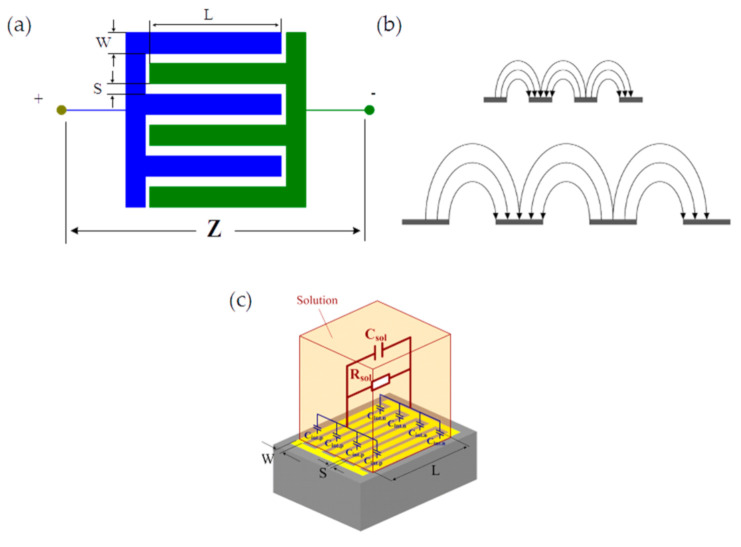
(**a**) Geometric parameters of interdigitated sensors; (**b**) Electric current displacement between electrodes; (**c**) Electrical model of an interdigitated sensor and sample (an ionic solution).

An IDT sensor is composed of two comb-like electrodes deposited on an insulated substrate. Each electrode’s digits have a width *W* and a length *L*, and there is a gap *S* between digits [21,22], as presented in Figure 1a. This specific electrode configuration produces an elliptic current displacement inside the sample, as shown in Figure 1b. Changes in *W* and *S* allow the electrical penetration depth in the sample to be adjusted. Ninety-five percent of the electric excitation signal power is concentrated within 2(*S + W*). This makes IDT sensors perfectly suitable for low-surface sample characterizations of, for instance, cell cultures or small-volume samples. An electrical equivalent model for an IDT sensor loaded with an electrolyte is presented in Figure 1c. The electrolyte is generally used as a simple reference medium for impedance-based sensor characterization.

According to Olthuis et al. [23], *R_sol_* and *C_sol_*, the resistance and capacitance of the ionic solution, can be linked via Equation (1) to electrolyte conductivity and permittivity using a cell factor *K_cell_*. This factor can be calculated as a function of IDT geometries using Equation (2).

(1) $$R_{sol}=\frac{K_{cell}}{\sigma_{sol}};\;\;C_{sol}=\frac{\varepsilon_{0}\varepsilon_{r,sol}}{K_{cell}},$$

(2) $$K_{cell}=\frac{2}{L\left(N-1\right)}\frac{K\left(k\right)}{K\left(\sqrt{1-k²}\right)},$$

where *σ_sol_* is the electrolyte conductivity (S·m^−1^), *ε_sol_* is the electrolyte relative permittivity, *N* is the number of digits, *L* is the digit length (m), *W* is the digit width (m), *S* is the space between two digits (m), and *K_cell_* is the cell factor (m^−1^). The function *K*(*k*) is the incomplete integral of the first module *k*, and is calculated with Equation (3). The metalization ratio is defined by *α = W/*(*S + W*).

(3) $$K\left(k\right)=\overset{1}{\underset{0}{\int }}\frac{1}{\sqrt{\left(1-t²\right)\left(1-k²t²\right)}}dt\;with\;k=\cos\left(\frac{\pi }{2}\frac{W}{S+W}\right).$$

where *σ_sol_* is the electrolyte conductivity (S·m^−1^), *ε_sol_* is the electrolyte relative permittivity, *N* is the number of digits, *L* is the digit length (m), *W* is the digit width (m), *S* is the space between two digits (m), and *K_cell_* is the cell factor (m^−1^). The function *K*(*k*) is the incomplete integral of the first module *k*, and is calculated with Equation (3). This function is used to formulate the elliptic electric field distribution, as described above. The metalization ratio is defined by *α = W*/(*S + W*).

### 2.2. Double Layer Impedance

**Figure 2:**
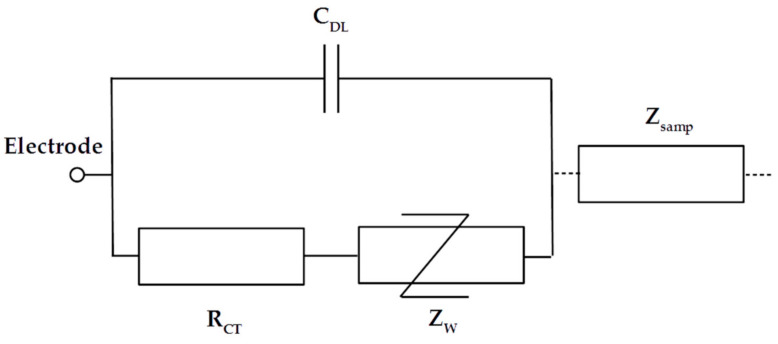
Electrical equivalent circuit for the double-layer impedance of one electrode.

The double-layer impedance represents the interface effects that occur when the polarized electrodes are in contact with an electrolyte. The double layer corresponds to two parallel layers of charge on a thin section (nanometric scale) of the electrode surface. These effects act as a barrier for low- frequency measurements and need to be taken into account in global modeling. Generally, it is necessary to limit these effects to increase the bandwidth of interest. A typical model for interface effects is composed of three elements making up an equivalent circuit, as presented in Figure 2. The charge transfer resistance *R_CT_* represents the resistive effect, and the double-layer capacitance *C_DL_* represents the capacitive effect. The Warburg impedance *Z_W_* with a constant phase (−π/2) is used to model the non-linear decrease in impedance induced by the diffusion of ionic species. These elements can be very difficult to model for complex electrolytes, such as blood plasma. Moreover, when the frequency is high enough, the decrease in *C_DL_*, the impedance short-circuit *R_CT_* and *Z_w_*, and the interface impedance become negligible compared to the impedance of the sample. Hence, double-layer impedance is generally only modeled by *C_DL_* when only the sample impedance is studied. This capacitance effect is proportional to the electrode surface and captured as the surface capacitance *C*_0_, with typical values ranging from 10 to 50 µF/cm^2^. For interdigitated sensors, global induced capacitance *C_interface_* can be calculated with the unitary digit capacitances *C_int,p_* and *C_int,n_* using Equations (4)–(6).

(4) $$C_{int,p}=C_{int,n}=LWC_{0},$$

(5) $$C_{interface}=\frac{N}{4}LWC_{0},$$

(6) $$Z_{interface}=\frac{1}{j\omega C_{interface}},$$

where *C_int,p_* and *C_int,n_* are the capacitances at each electrode digits (F), *C_interface_* is the global capacitance at the sensor (F), *Z_interface_* is the induced impedance in series with the sample impedance (Ω), and *C*_0_ is the surface capacitance at the interface electrode/electrolyte (F/m^−1^).

### 2.3. Sample Impedance

In a linear, homogeneous, and isotropic samples, measured impedance *Z_samp_* (Ω) and admittance *Y_samp_* (S) depend on the sample’s intrinsic properties (conductivity *σ_samp_* and permittivity *ε_samp_*) and the sensor factor *K_cell_*, as seen in Equation (7) [24]. *Z_samp_* is the sample’s impedance and does not take into account other effects, such as double-layer capacitance.

(7) $$Z_{samp}=\frac{K_{cell}}{\sigma_{samp}+j\omega \varepsilon_{0}\varepsilon_{r,samp}}\;\to Y_{samp}=\frac{\sigma_{samp}+j\omega \varepsilon_{0}\varepsilon_{r,samp}}{K_{cell}}\to \left\{\begin{array}{c} G_{samp}=\frac{\sigma_{samp}}{K_{cell}}\\ C_{samp}=\frac{\varepsilon_{0}\varepsilon_{r,samp}}{K_{cell}} \end{array}\right..$$

**Figure 3:**
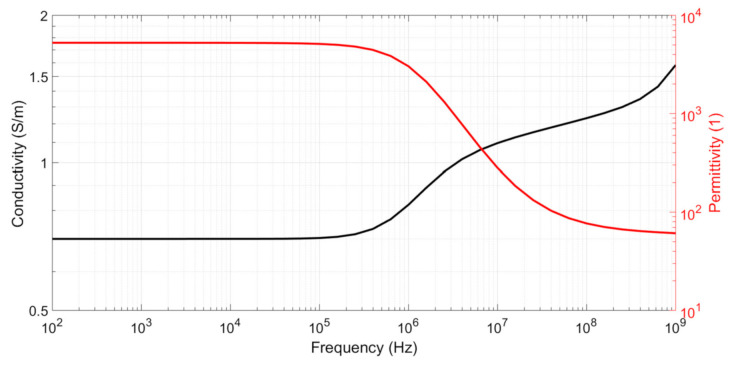
Typical complex conductivity and relative permittivity values for a blood sample.

For a simple sample, such as that of an electrolyte, *σ_samp_* and *ε_r,__samp_* can be considered constants. For more complex biological samples, such as blood, these values depend on frequency and are referred to as complex conductivity *σ_samp_*(*ω*) and complex relative permittivity *ε_r,__samp_*(*ω*), respectively. Figure 3 shows the typical values for the complex conductivity and relative permittivity of blood [12]. The two levels of relative permittivity represent the effects of cell membrane capacitance and water permittivity, respectively. The two levels of conductivity represent the effects of extracellular medium conductivity and the combination of extracellular/cytoplasm conductivity, respectively. The passage from the first to second level is known as the *β* dispersion and occurs when the impedance of the capacitive cell membrane becomes negligible compared to the cytoplasmic impedance. The second increasing of conductivity is called the *γ* dispersion and appears at microwave frequencies (GHz). It corresponds to water molecular excitation and is not studied here. The blood conductivity diagram shows the necessity of performing the conductivity extraction correctly before and after *β* dispersion when characterizing a biological sample. In the case of a blood sample, this dispersion begins at approximately 250 kHz.

After adding the interface effect in series with the samples, the global measured impedance becomes

(8) $$\left\{\begin{array}{c} G_{Tot}\left(\omega \right)=\frac{\omega^{2}G_{samp}C_{interface}^{2}}{G_{samp}^{2}+\omega^{2}{\left(C_{samp}+C_{interface}\right)}^{2}}\\ C_{Tot}\left(\omega \right)=\frac{C_{interface}G_{samp}^{2}+\omega^{2}C_{samp}C_{interface}\left(C_{samp}+C_{interface}\right)}{G_{samp}^{2}+\omega^{2}{\left(C_{samp}+C_{interface}\right)}^{2}} \end{array}\right..$$

(9) $$\underset{\omega \to 0}{\lim}C_{Tot}=C_{interface}$$

**Figure 4:**
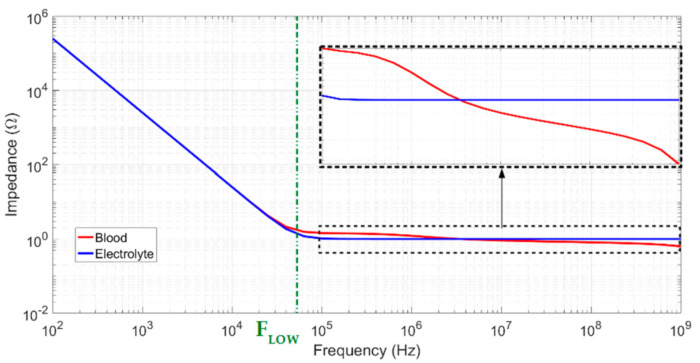
Typical curve profiles for biological and electrolyte samples.

*G_Tot_* and *C_Tot_* correspond to global measured conductance and capacitance induced by the sample and interface impedances, respectively. At low frequencies, the global capacitance tends to *C_interface_*, as presented in Equation (9). When the frequency increased, *Z_interface_* became negligible compared to *Z_samp_*, and total impedance can be considered equal to *Z_samp_*. The typical curve profiles for a biological sample and an electrolytic sample, showing the double-layer capacitance effect, are shown in Figure 4. Two plateaus are clearly visible and correspond to blood conductivity. If the lower cutoff frequency is too high, the first plateau can be completely hidden by double-layer impedance and interfere with the measurement. The low cutoff frequency is defined in Equation (10) below via an analogy with low and high passive first-order filters using the conductivity of the medium.

(10) $$f_{low}=\frac{1}{2\pi R_{samp}C_{interface}}\;when\;f=\;f_{low}\;,\;|Y_{samp}|=\;\frac{G_{samp}}{\sqrt{2}}\;and\;|Z_{samp}|=\sqrt{2}\;R_{samp}$$

Since *Z_interface_* is not purely capacitive, the first relation in Equation (10) may prove difficult to use. Hence, the second equation is usually preferred. In this work, this second equation was used to determine *f_low_* from the measured impedance spectrum. *R_samp_* and *G_samp_* can be extracted from the real part of the impedance or admittance when a plateau is visible in the spectrum (predominance of resistive/conductive effects). These parameters are extracted from the real part of the impedance measured in the center of each plateau. Furthermore, *σ_samp_* can be calculated using the extracted *R_samp_* or *G_samp_* and Equation (7).

## 3. Sensor Optimization

As mentioned above, the interface impedance acts as a barrier at low frequencies. Optimizing the frequency band consists of reducing the *f_low_* value. Since *f_low_* depends on both the sample properties and sensor geometry, it is possible to optimize the frequency band by optimizing the sensor design. Equation (10) was developed by using Equations (1), (2) and (5) to check which geometric parameter was the most suitable one to study. Hence, Equation (11) is obtained. As *σ_sol_* and *C*_0_ depend on sample properties, decreasing *f_low_* is equivalent to decrease the term given in expression (12). It can be seen that this term depends on *N*, *W*, and *S*. In addition, the term (*N* − 1)/*N* will be negligible for large *N*.

(11) $$f_{low}=\frac{1}{\pi }\frac{\sigma_{samp}}{C_{0}}\frac{\left(N-1\right)}{N}\frac{1}{W}\frac{K\left(\sqrt{1-k²}\right)}{K\left(k\right)}$$

(12) $$\frac{\left(N-1\right)}{N}\frac{1}{W}\frac{K\left(\sqrt{1-k²}\right)}{K\left(k\right)}$$

**Figure 5:**
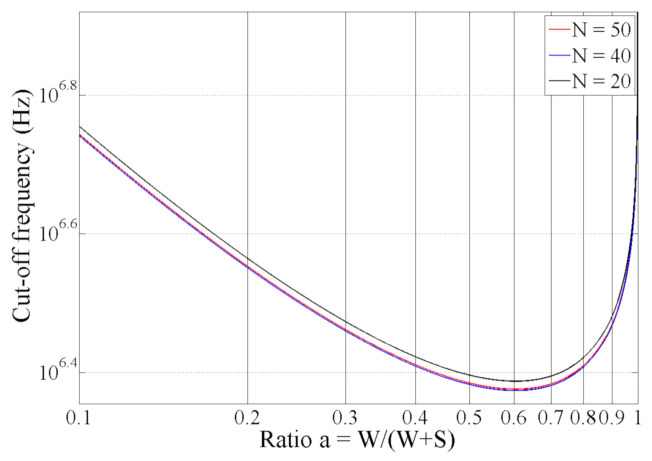
Low cutoff frequency as a function of *α*.

Only *W* and *S* seem to have a significant impact on *f_low_* through the *K_cell_* and “*1/W*” terms. It is relevant to study the optimization of *α*. Clearly, *f_low_* could be reduced by increasing *W*, but doing so implies an increase in sensor size as well as the measurement volume: the smaller the cell constant (large electrodes), the lower the cut-off frequency, but the faster the measurement volume will increase. Here, we are interested in optimizing the sensor for a given volume/measurement surface on a microscopic scale. (*W + S*) and *N* were set to constant values to maintain the same volume for the investigation. Analytical simulations were performed by varying the *α* ratio from 0.1 to 1 and holding the other parameters constant. *L* was set to 2 mm, and (*W + S*) was set to 50 µm. Finally, *σ_samp_* was set to 0.6 S/m (conductivity of blood at low frequency), and *C*_0_ was set to 40 µF/cm^2^. Three values of *N* (20, 40 and 50) were tested. The results are shown in Figure 5. These results demonstrate that the value of *N* does not have a significant impact on the cutoff frequency. In contrast, the value of *f_low_* mainly depends on *α* and is a minimum for *α* = 0.6. These results show that it is possible to decrease the low cutoff frequency by a factor up to approximately 2.5 by optimizing the metalization ratio *α*.

## 4. Sensor Realization

### 4.1. Sensor Manufacturing

**Figure 6:**
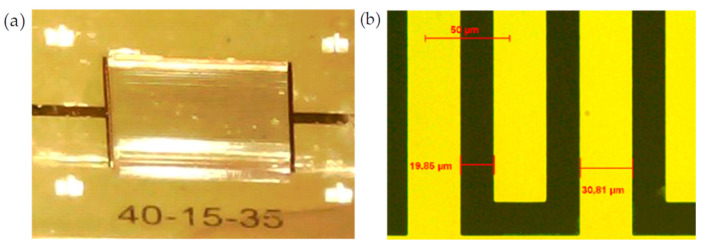
(**a**) Photograph of Sensors 1 with SU_8 well and blood sample, and (**b**) optical microscope image of different Sensor 1 digits.

To prove our assumptions, three sensors were realized in a clean room using standard microfabrication techniques. Platinum electrodes were structured via sputtering deposition and optical lithography on a glass substrate. Wells were created by using negative SU_8 resin with a thickness of approximately 400 µm. Sensor 1 is illustrated in Figure 6. All sensors presented the same periodicity, electrode number and electrode length. Different *α* ratios were tested to prove our assumptions. The sensor geometries are listed in Table 1. All sensors presented the same pitch (*W* + *S*) and the same surface of 2 × 2 mm^2^ for ease of comparison. The pitch was set to 50 µm to provide a penetration depth of only several cell sizes (from 6 µm to 15 µm for red blood cells and white blood cells, respectively). The smallest *W* and *S* values were of the same size order as blood cells. These dimensions allowed increased sensitivity by performing characterizations of a few cell layers in depth.

### 4.2. Reference Measurements

**Figure 7:**
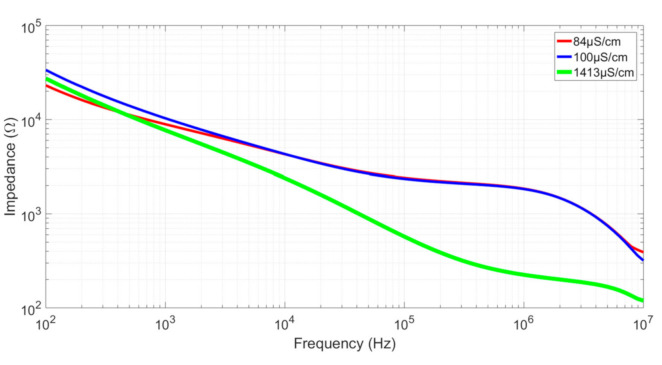
Bode diagrams of the impedances for three different calibrated solutions measured with Sensor 1.

To verify both the validity of our model and the integrity of the sensors, measurements were performed for three different calibrated solutions of 84 µS/cm, 100 µS/cm, and 1413 µS/cm. All measurements were performed by depositing a 2 µL drop into the well with a micropipette. Examples of the resulting impedance diagrams are shown in Figure 7 for Sensor 1. All results are reported in Table 2. The *K_cell_* factor was calculated for *R_sol_* on the curves’ plateaus using Equation (1). The calculated values of *K_cell_* were in accordance with the simulated ones. Sensor 3 had a higher error in *K_cell_* determination compared to the others, which can be explained by the fact this sensor has the smallest useful gap size (*S* = 10 µm). Using optical lithography, the resolution is approximately 0.5 µm for each edge, or 1 µm for a digit, representing a possible error of 10% for a 10 µm digit, which is close to the *K_cell_* error for Sensor 3. Note that Sensor 1, with *α* = 0.6, presents the smallest cutoff frequencies, as predicted in the “Sensor optimization” section. The capacitance effect observed in the higher frequencies can be attributed to the experimental setup limitations, including the capacitance induced by the interfacing Printed Circuit Board (PCB), the connections, and the measurement devices.

## 5. Blood Characterization

### 5.1. Experimental Setup

**Figure 8:**
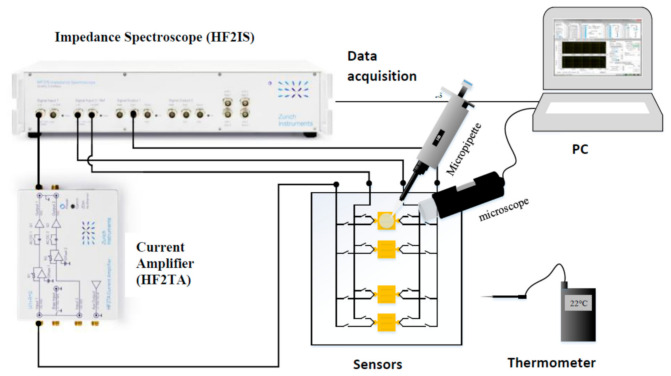
Bio impedance measurement setup.

**Figure 9:**
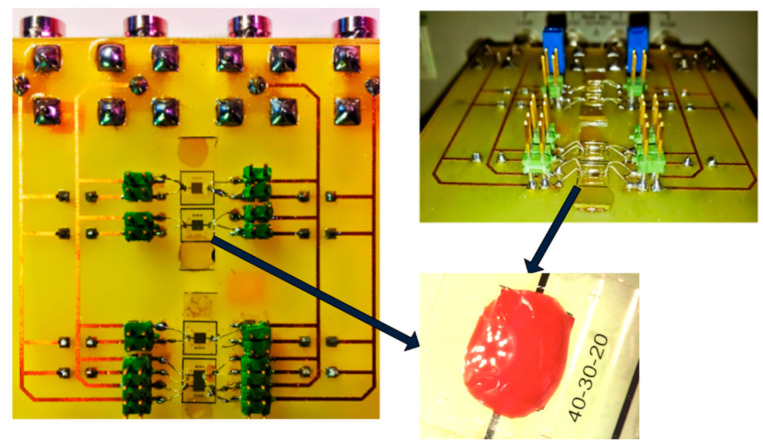
Interdigital electrode sensors connected to the PCB circuit.

Figure 8 represents a general view of the instrumentation setup developed in this work for the electrical characterization of biological media. This instrumentation setup consists of the following elements:

(a) Biofluid samples placed directly on the sensor (Figure 9).
(b) A microscope to observe the position of the volume of liquid.
(c) A thermometer to measure the ambient temperature.
(d) A micropipette (Socorex Micropipette Acura 825)
(e) HF2TA current amplifier (manufactured by Zurich Instrument),
(f) HF2IS impedance spectroscope for the frequency range from 0.7 μHz to 50 MHz.
(g) A computer for observing and processing measurement data using LabVIEW^®^ application.

This application allows us to enter the measurement parameters described above and save the values of the impedance spectra as text files.

The optical microscope allows us to correctly place the drop of the biofluid on the sensor. The interface card containing the sensors (Figure 9) is connected to the HF2IS impedance analyzer and to the HF2TA current amplifier via short SMA cables in order to reduce the length of the measurement loop as much as possible. The impedance spectroscope is connected via a USB cable to a computer to control it and retrieve the data.

### 5.2. Sample Preparation

Blood sampling was performed under medical supervision (University Hospital, CHRU Nancy, France) using the same donor. The samples were placed into three 6 mL tubes with heparin. Measurements were taken within 10 min of sampling.

To ensure good repeatability for the measurements of all sensors, each sample was deposited on each sensor using the following procedure:

- The tube was shaken slightly for 1 min before sampling with a micropipette.
- A 2 µL sample was obtained with an adjustable micropipette and deposited into the well (Figure 9). This volume was chosen to ensure that all the sensor cavities were full. As described in Section 2.1, only the first 100 µm of thickness of the sample in contact with the IDT was really due to the small penetration depth of the IDT sensors, ensuring that measurements will not be impaired by round-shaped droplets, the sample surface in contact with the air or cavity walls, or the sample rising above the cavity.
- The impedance spectrum acquisition started 10 s after sample deposition. The impedance measurement was then performed within several seconds at 1 V sinus amplitude.
- The room temperature was maintained at 25 ± 1 °C during the measurement campaign.

### 5.3. Results

**Figure 10:**
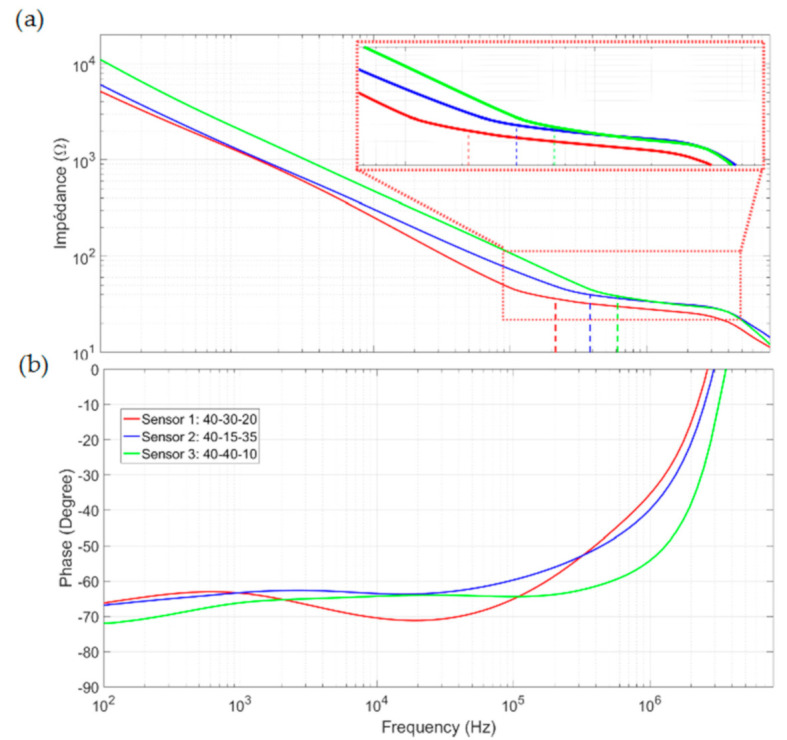
Bode diagrams for blood impedance characterizations (**a**) in modules, and (**b**) in phases.

Measurements were performed using the experimental setup described in Section 5.1 and the sampling procedure described above for each sensor. The results are shown in Figure 10 in the form of Bode diagrams for the modules and phases. *f_low_* represents the measured low-frequency cut-off using the method proposed in Section 2.3. As in the measurements of the calibrated solution, Sensor 1 has the smallest low-frequency cut-off and the widest bandwidth. Unlike the two other sensors, two plateaus are visible from and after the cutoff frequency for sensor 1. This result proves that only sensor 1 is able to characterize the complete spectrum for blood impedance. For the other sensors, the double-layer impedance remains non-negligible until the cutoff frequency and interferes with sample impedance measurement. 

Low-frequency blood conductivity was calculated using the *R_sol_* values measured on the plateaus just after the *f_low_* values and Equation (1). The results extracted from the impedance spectrum are summarized in Table 3. The values 0.69, 0.89, and 0.43 S·m^−1^ were obtained for sensors 1, 2, and 3, respectively. These conductivities are of the same order as the 0.7 S/m value obtained by Gabriel [12]. The difference between sensor 1 and the other two sensors can be explained by its lower immunity to the interface effect that disturbs the *R_sol_* measurement and conductivity extraction. For sensor 1, *f_low_* is slightly lower than at the beginning of the *β* dispersion band studied in the theoretical section (215 kHz, down from 250 kHz) and allows the first plateau of conductivity to be measured. For the other sensors, the measurements were performed away from this plateau and are incorrect.

## 6. Conclusions

An analytical model for an IDT sensor was proposed. Using this model, the effect of geometric parameters on global impedance was studied in order to propose an optimized sensor for blood characterization. It appears that the number of electrodes *N* and digit length *L* do not contribute significantly to improving sensor bandwidth. To the contrary, the metalization ratio *α*, which depends only on the digit width *W* and gap *S*, is a relevant parameter for optimizing the sensor. The analytical simulations showed that the best optimization is obtained for *α* = 0.6. This *α* value permits the low cut-off frequency to be reduced by a factor of up to 2.5. To validate our theoretical results, measurements were performed on three different sensors using the same active surface area (2 mm × 2 mm) but different ratios *α*. The results obtained with the calibrated solutions proved the validity of our model and the possibility improving sensor bandwidth. Finally, blood sample characterizations were performed using the sensors. The optimized sensor was able to characterize the blood sample and extract its intrinsic property (electrical conductivity), achieving good concordance with the reference blood conductivity provided in the literature.

## Figures and Tables

**Table 1:** Sensor geometries.

Sensor Number	N	L (µm)	W (µm)	S (µm)	Theoretical *K_cell_* (m^−1^)	*α* Ratio
1	40	2000	30	20	21.8	0.6
2	40	2000	15	35	35.8	0.3
3	40	2000	40	10	15.1	0.8

**Table 2:** Sensor results for the calibrated solutions.

Sensor Number	Theoretical *K_cell_* (m^−1^)	*α* Ratio		84 µS/cm		100 µS/cm			1413 µS/cm		
			fc,L (kHz)	*K_cell_*	*K_cell_* Error (%)	fc,L (kHz)	*K_cell_*	*K_cell_* Error (%)	fc,L (kHz)	*K_cell_*	*K_cell_* Error (%)
1	21.8	0.6	75.6	22.3	2.3	85	22.01	0.96	433	22.65	3.9
2	35.8	0.3	170	35.49	0.87	486	34.99	2.26	1960	35.55	0.7
3	15.1	0.8	135	16.83	11.48	305	16.08	6.49	2400	17	12.57

**Table 3:** Sensor results for the calibrated solution.

Sensor Number	N	L (µm)	W (µm)	S (µm)	*f_low_* (kHz)	Measured *K_cell_* (m^−1^)	*α*	*R_sol_* (Ω)	*σ_b__lood_* (S/m)
1	40	2000	30	20	215	22.32	0.6	32.35	0.69
2	40	2000	15	35	358	35.834	0.3	40.26	0.89
3	40	2000	40	10	614	16.64	0.8	38.70	0.43
